# Total elbow arthroplasty in rheumatoid arthritis: A population-based study from the Finnish Arthroplasty Register

**DOI:** 10.3109/17453670903110642

**Published:** 2009-08-01

**Authors:** Eerik T Skyttä, Antti Eskelinen, Pekka Paavolainen, Mikko Ikävalko, Ville Remes

**Affiliations:** ^1^Department of Orthopedics and Traumatology, Peijas Hospital, Helsinki University Central HospitalHUS, FI-00029Finland; ^2^COXA Hospital for Joint ReplacementTampere, 33101Finland; ^3^ORTON Orthopedic HospitalHelsinki, FI-00281Finland; ^4^Rheumatism Foundation HospitalHeinola, FI-18120Finland; ^5^Department of Musculoskeletal Medicine, Medical School, University of TampereTampereen Yliopisto, FI-33014Finland

## Abstract

**Background and purpose** Although total elbow arthroplasty (TEA) is a recognized procedure for the treatment of the painful arthritic elbow, the choice of implant is still obscure. We evaluated the survival of different TEA designs and factors associated with survival using data from a nationwide arthroplasty register.

**Methods** 1,457 primary TEAs for rheumatoid elbow destruction were performed during 1982 to 2006 in one hospital specialized in the treatment of rheumatoid arthritis (n = 776) and in 19 other hospitals (n = 681). The mean age of the patients was 59 years and 87% of the TEAs were performed in women. We selected different contemporary TEA designs, each used in more than 40 operations including the Souter-Strathclyde (n = 912), i.B.P./Kudo (n = 218), Coonrad-Morrey (n = 164), and NESimplavit/Norway (n = 63) to assess their individual survival rates. Kaplan-Meier analysis and the Cox regression model were used for survival analysis.

**Results** The most frequent reason for revision was aseptic loosening (47%). We found no differences in survival rates between different TEA designs. We did, however, find a 1.5-fold (95% CI: 1.1–2.1) elevated risk of revision in unspecialized hospitals as compared to the one hospital specialized in treatment of rheumatoid arthritis. In the Souter-Strathclyde subgroup, there was a reduced risk of revision (RR 0.6, p = 0.001) in TEAs implanted over 1994–2006 as compared to those implanted earlier (1982–1993). The 10-year survivorship for the whole TEA cohort was 83% (95% CI: 81–86), which agrees with earlier reports.

**Interpretation** The influence of implant choice on the survival of TEA is minor compared to hip and knee arthroplasties. Inferior survival rates of the TEAs performed in the unspecialized hospitals demonstrates the importance of proper indications, surgical technique, and postoperative follow-up, and endorses the need for centralization of these operations at specialized units.

## Introduction

The elbow is involved in two-thirds of all patients with rheumatoid arthritis (RA), and, in joints with severe destruction, total elbow arthroplasty (TEA) is often indicated (Scott et al. 1986, Hämäläinen et al. 1991, Lehtinen et al. 2001). There are several unlinked and linked TEA designs currently available with radically different geometries (King et al. 1993, Kamineni et al. 2005). TEA is, however, much less common than hip or knee replacement; it has an annual incidence of 1 per 100,000 inhabitants (Rahme et al. 2001) and comprehensive assessment of different concepts and models is thus difficult in comparative randomized studies.

Recent studies have indicated that observational reviews may give results that are similar to those of randomized trials (Benson and Hartz 2000, Concato et al. 2000). However, any generalization of results from such observational studies must be made with caution, since TEA is a relatively specialized orthopedic procedure and is frequently performed by only a few individuals. The monitoring of arthroplasty by means of long-established nationwide arthroplasty registers has improved the quality of hip replacement (Herberts and Malchau 2000); these registers have proved to be valuable tools in the evaluation of concepts in joint replacement surgery (Eskelinen et al. 2005). The Finnish Arthroplasty Register was established in 1980, and data from hip, knee, shoulder, and elbow replacements have been continuously recorded for more than 25 years (Paavolainen et al. 1991).

The present study was initiated in order to examine—at a nationwide level—the survival of different TEA designs and the factors affecting survival in patients with RA, by using the data from the Finnish Arthroplasty Register.

## Patients and methods

This study was based on information recorded in the Finnish Arthroplasty Register (Puolakka et al. 2001) relating to patients who underwent TEA between 1982 and 2006. The register contains data on 1,612 primary TEAs, each of which was recorded individually for every operation since the start of the register. Of these 1,612 TEAs, 1,457 (90%) were performed due to RA and these were selected for further analysis. The coverage of the Finnish Arthroplasty Register was analyzed in 1994–1995 by comparing its data with those of the discharge registers of the participating hospitals; it was found to cover 90% of implantations and implant removals. Since 1995, every few years the data in the register have been compared with those in the hospital discharge registers. Currently, over 95% of implantations are recorded. An English translation of the form used for data collection has been published elsewhere (Paavolainen et al. 1991). Revisions were linked to the primary operation using the unique personal identification number assigned to each resident of Finland.

### Hospital-specific trends

In Finland, the majority of the 1,457 TEAs for RA have been performed in a foundation-based hospital specialized in the treatment of RA (Table 1). Nineteen other hospitals have also performed TEAs for RA. We analyzed the overall survival of TEAs performed in a specialized hospital and compared it to the unspecialized hospitals group in order to assess the effect of hospital volume. This comparison was only performed for the Souter-Strathclyde subgroup, as only 27 TEAs had been performed in the specialized hospital with other designs of total elbow replacement (Table 1).

**Table 1.** Trends in total elbow arthroplasty (TEA) for rheumatoid arthritis in one hospital specialized in the treatment of rheumatoid arthritis and in 19 other hospitals in Finland during 1982–2006

	Specialized hospital	Other hospitals
Mean follow-up (SD)	6.3 (4.3)	8.8 (5.0)
Total number of TEAs	776	681
TEAs during 1982–1993	295	102
TEAs during 1994–2006	460	578
NESimplant/Norway	27	36
Coonrad-Morrey	0	164
i.B.P./Kudo	0	218
Souter-Strathclyde	712	200

### Implant-specific trends

Inclusion criteria In order to assess the survival of different TEA designs, we selected only those designs that had been used in more than 40 operations during the study period (Havelin et al. 1995). In addition, only implants with a mean follow-up of more than 3 years, and with more than 20 patients at risk at five years, were included. We included bilateral TEAs as separate cases in the analysis since bias by this procedure is likely to be negligible (Robertsson and Ranstam 2003).

### Types of prostheses selected

To meet our inclusion criteria, the following implants were selected: NESimplavit (Implantcast, Buxtehude, Germany), formerly Norway elbow (3M, St. Paul, MN); Kudo and i.B.P. (Biomet, Warsaw, IN), Souter-Strathclyde (Stryker, Kalamazoo, MI), and Coonrad-Morrey (Zimmer, Warsaw, IN) (Table 2). All the Souter-Strathclyde models (short and long-stemmed humeral components and metal-backed and all-polyethylene ulnar components) were considered as one group. Since this prosthesis has been used in 2 out of 3 of all TEAs performed in Finland, it was used as the reference design for purposes of comparison. The NESimplavit is a new brand name that formerly went under the name of Norway elbow prosthesis. These are identical in design, which allowed us to pool the data. All the implanted Kudo prostheses were of the latest generation, the i.B.P. being the latest modified version. In order to be able to compare the results of these unconstrained resurfacing models with other designs, the data from these two models were also pooled. All of the Coonrad-Morrey models were also considered as one, even though small adjustments have been made to the metal composition, surface coating, and locking pin. Time-dependent trends were also analyzed for all implants and for the Souter-Strathclyde prosthesis alone. Of the contemporary implant designs, only the Discovery elbow (Biomet) was excluded due to an insufficient number of operations.

**Table 2.** Different prosthesis designs used in total elbow arthroplasty for rheumatoid arthritis from 1982 through 2006

Implant	n	Concept	Mean age	Women	Implantation period	Number of hospitals	Operations per hospital (range)
NESimplant/Norway	63	Unconstrained, humero-ulnar relationship realigned	60	86%	1997–2006	4	11 (4–15)
Coonrad-Morrey	164	Linked, semicontrained “sloppy” hinge	63	92%	1995–2006	14	10 (1–58)
i.B.P./Kudo	218	Unconstrained, anatomic humero-ulnar relationship	60	79%	1994–2006	13	16 (1–56)
Souter-Strathclyde	912	Unconstrained, humero-ulnar relationship realigned	58	87%	1982–2006	12	75 (1–697)^a^

^a^ 78% of the Souter-Strathclyde total elbow arthroplasties were performed in one hospital specialized in the treatment of rheumatoid arthritis.

### Statistics

The endpoint for survival was defined as revision involving either one component or the whole implant (removal or exchange). Kaplan-Meier survival data were used to construct the survival probabilities of implants at 4, 7, 10, and 15 years. Survival data obtained in the Kaplan-Meier analysis were compared by the log-rank test. The Cox multiple-regression model was used to study differences between groups and to adjust for potential confounding factors. In all models, the confounding factors were age and gender. The factors studied with the Cox model were as follows: TEA designs, hospital type (specialized hospital vs. all other hospitals), and implantation period. All models included adjustment for differences in age and gender.

Survivorships of the other TEA designs were compared with that of the Souter-Strathclyde TEA (reference design). As the Souter-Strathclyde was the only design that had been used over 1982–1993 (Table 2), the effect of the implantation period (cohort effect analysis; see Eskelinen et al. 2005) was only analyzed in the Souter-Strathclyde subgroup and for 2 time periods: 1982–1993 and 1994–2006. In order to minimize the effects of the confounding factors, time period analysis was also adjusted for hospital type. The Cox regression analyses provided estimates of survival probabilities and revision risk ratios (RRs) for different factors. Estimates from the Cox analyses were used to construct adjusted survival curves at mean values of the risk factors. The Wald test was used to calculate p-values for data obtained from the Cox multiple regression analysis. Differences between groups were considered statistically significant if the p-values were less than 0.05 in a two-tailed test. We used SPSS statistical software version 14.0.

## Results

### Patient characteristics

Of the 1,457 TEA operations, 1,262 (87%) were performed in women patients. At the time of the operation, the mean age of the patients was 59 (18–89) years. 793 (54%) of all the TEAs were performed on the right elbow. The mean annual insidence of TEA was 1.3 per 100,000 inhabitants.

### Implants

Over the whole study period, 9 different TEA designs were used, 6 of them in 4 groups with more than 40 operations (Table 3). All implants were cemented. At the end of the study period (2005–2006), 6 TEA designs were still in use in Finland. Of the models still being used, only the Discovery elbow (Biomet) was excluded due to an insufficient number of operations. Epidemiological data relating to the designs analyzed are given in Table 2.

**Table 3.** Implants used for total elbow arthroplasty in rheumatoid arthritis in Finland from 1982 through 2006 and their inclusion in the study

Brand of implant	n	Inclusion in analysis	Used during years
Souter-Strathclyde	912	Yes	1982–2006
Kudo	175	Yes	1994–2003
Coonrad-Morrey	164	Yes	1995–2006
i.B.P.	43	Yes	2001–2006
Norway	13	Yes	1997–1998
Pritchard-Walker Mark II	20	No	1987–1998
NESimplant	50	Yes	2003–2006
Discovery	52	No	2003–2006
Other	1	No	1982
Total	1,345		

### Hospital-specific trends

**Figure 1.**
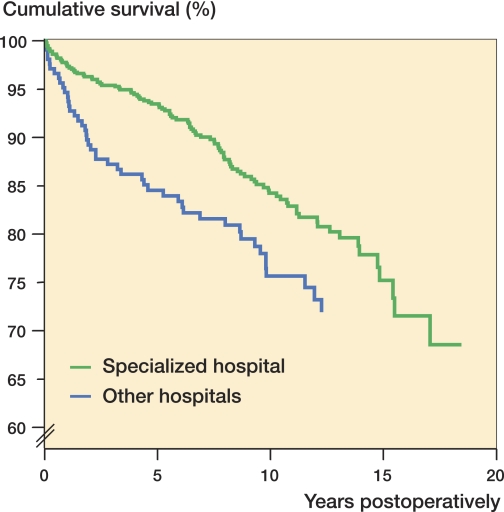
Cox-adjusted cumulative survival of TEAs for rheumatoid arthritis in one hospital specialized in the treatment of rheumatoid arthritis (n = 776) and in 19 other hospitals (n = 681) in Finland from 1982 through 2006. The endpoint was defined as revision for any reason. Adjustment was made for age, sex, and prosthesis design.

In the Souter-Strathclyde subgroup, the Cox regression model (adjusted for age and sex) showed a 1.5-fold (95% CI: 1.1–2.2) increased risk of revision for the non-specialized hospitals as compared to the specialized hospital (p = 0.02) (Figure 1).

### Survival of TEA designs

**Figure 2.**
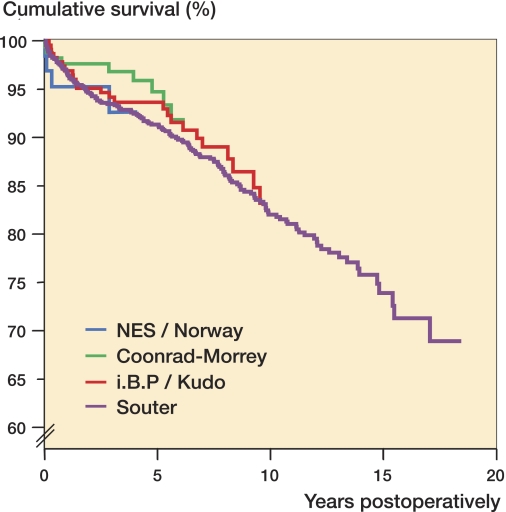
Cox-adjusted cumulative survival of different prosthesis designs used in TEA for rheumatoid arthritis in Finland from 1982 through 2006. The endpoint was defined as revision for any reason. Adjustment was made for age and sex.

We found no differences in survival rates between different TEA designs over the whole study period, using either the Kaplan-Meier analysis or the Cox regression model (with or without adjustment for age and sex) (Table 4 and Figure 2).

**Table 4.** Cox-adjusted survival of different total elbow replacement designs used in patients with rheumatoid arthritis from 1982 through 2006 in Finland

A	B	C	D	E	F	G	H	I	J	K	L	M
NES/Norway	63	3.5 (0–10)	29	92 (85–100)	0	–	0	–	0	–	1.1 (0.4–2.7)	0.9
Coonrad-Morrey	164	4.6 (0–10)	108	96 (92–99)	36	89 (83–96)	3	–	0	–	0.7 (0.3–1.3)	0.2
i.B.P./Kudo	218	6.5 (0–13)	173	93 (90–97)	110	89 (84–94)	46	83 (76–90)	0	–	0.9 (0.3–1.3)	0.5
Souter-Strathclyde	912	8.8 (0–25)	771	93 (91–94)	612	88 (86–90)	419	82 (80–85)	97	75 (70–79)	1.0	–

A Brand of implant

B n

C Mean follow (range), years

D Number of elbows at risk at 4 years

E 4-year survival (95% CI), percent

F Number of elbows at risk at 7 years

G 7-year survival (95% CI), percent

H Number of elbows at risk at 10 years

I 10-year survival (95% CI), percent

J Number of elbows at risk at 15 years

K 15-year survival (95% CI), percent

L Adjusted RR for revision (95% CI) from the Cox regression analysis (other TEA designs compared to the Souter-Strathclyde prosthesis; adjustment was made for age and sex).

M p-value

### Cohort effect analysis

**Figure 3.**
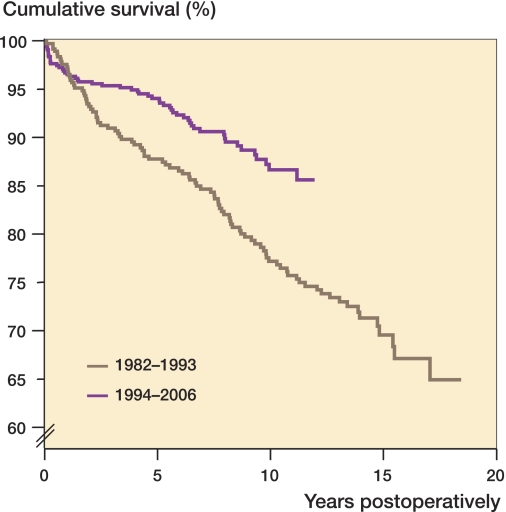
Cox-adjusted cumulative survival of the Souter-Strathclyde total elbow replacements used for rheumatoid arthritis in Finland over 2 different time periods. Adjustment was made for age, sex, and type of hospital.

In the Cox regression analysis of the Souter-Strathclyde subgroup, there was a significantly reduced risk of revision (RR 0.6; 95% CI: 0.4–0.8) in TEAs implanted in 1994–2006 as compared to those implanted earlier, i.e. 1982–1993 (p = 0.001) (Figure 3).

### Effect of age and sex on survivorship

Age and sex did not have any statistically significant effect on survivorship in the Cox multiple regression model (unadjusted and adjusted for implant design)—either when all TEAs were analyzed or when analyzing the subgroup of patients treated with the Souter-Strathclyde total elbow replacement.

### Revision operations

During the period 1982–2006, 201 revisions were reported. Thus, the 10-year survivorship for the whole TEA cohort was 83% (95% CI: 81–86). The most common reason for revision was aseptic loosening (47%, n = 95). This was followed by prosthesis dislocation (16%, n = 32), periprosthetic fracture (14%, n = 29), infection (12%, n = 25), fracture of the prosthesis (4%, n = 9), and malalignment of the prosthesis (1%, n = 2). Other, miscellaneous reasons (including exchange of the liner) accounted for 4% of the revisions (n = 9).

## Discussion

To the best of our knowledge, there have been no previous reports on results of TEA conducted at a nationwide level. The mean annual incidence of TEA was 1.3 per 100,000. We found no differences in survival between different TEA designs or concepts, based on the data recorded in the Finnish Arthroplasty Register. The most significant finding in our study was the better TEA survival when performed in a hospital that specialized in the treatment of rheumatoid arthritis.

We are aware that the current register-based study had certain limitations. For example, we were not able to report any subjective outcome measurements, e.g. Mayo Elbow Performance Score or disease-specific quality of life measurements. Moreover, it is not possible to conduct radiographic analyses in the large number of register-based patients. Furthermore, when rheumatoid patients are involved, a register-based study may have the pitfall that some of the patients diagnosed as having RA may actually be affected by juvenile arthritis or other subtypes of chronic arthritis. Little is known about TEA in different subtypes of chronic arthritis (Connor et al 1998).

The primary indication for TEA is a painful arthritic elbow with Larsen grade-IV or grade-V (Larsen et al. 1977) rheumatic destruction (Scott et al. 1986, Hämäläinen et al. 1991, Little et al. 2005a). There are only limited published data to guide a surgeon in implant selection. A recent systematic review of the English-language literature (Little et al. 2005a) found that a high proportion of the published studies on TEA had originated from the establishments of the designers of the implants. In addition, no recognized form of survival analysis such as the Kaplan-Meier technique had usually been used in these studies. In their attempt to re-calculate the revision rates for different TEA designs in patients with RA, Little et al. found an overall revision rate of 13% at 5 years. Our register-based study has revealed a similar rate of prosthesis survival, with 88–89% of patients revision-free at 7 years.

Traditionally, the TEA designs have been divided into 3 categories: fully constrained rigid-hinge design, semi-constrained hinge design, and unconstrained unlinked design. Fully constrained designs have a lack of flexibility in the coronal plane and in rotation, resulting in high shear stresses and early loosening rates, and these model are no longer in use (Garrett et al. 1977, Amis et al. 1981). Unconstrained models rely on sufficient bone stock and an intact soft tissue sleeve, whereas the semi-constrained designs can be used in an elbow that is unstable because of bone or soft tissue deficiency (Wright et al. 2000). There are, however, reports of favorable results after TEA with the use of the unconstrained Souter-Strathclyde implant for the severest forms of rheumatic destruction and substantial bone loss (Ikävalko et al. 2004). On the other hand, Kamineni and colleagues (2005) found large variation in intrinsic constraint of unlinked TEA designs. Thus, it may not be practical to consider all unlinked prostheses as a group. In their review article, Little and colleagues (2005a) found no differences in survival between different prostheses or concepts. In the studies comparing contemporary TEA designs, it has not been possible to demonstrate the superiority of one model or concept over another (Connor et al. 1998, Wright et al. 2000, Little et al. 2005b).

In the present study, we pooled the results of the Kudo prosthesis and its latest version (i.B.P.), and we also pooled the data for the NESimplavit and its former brand, the Norway prosthesis. In spite of this, we could not find statistically significant differences between survival rates of the different designs. In their comparative study of 3 implants using any revision as the endpoint, Little and coworkers (2005b) found the 5-year survival rates of the Souter-Strathclyde, Kudo, and Coonrad-Morrey implants to be 85%, 93%, and 90%. The differences were not statistically significant, however. Our survival rates (Table 3) are in line with their findings.

Several studies have found an association between the hospital volume and adverse events in the context of total hip arthroplasty (THA), but no such studies on TEA have been published. Both surgeon volume and hospital volume have been suggested to be the best indicators of orthopedic adverse events in patients undergoing THA (Solomon et al. 2002). In a systematic literature review, an association was found between higher hospital volumes and lower rates of mortality and hip dislocation (Shervin et al. 2007). Inferior survival rates of the TEAs performed in the unpecialized hospitals demonstrate the importance of proper indications, surgical technique, and postoperative follow-up, and endorse the centralization of these operations at specialized units.

The inferior survival rates in the Souter-Strathclyde TEAs implanted in the early years of the Finnish Arthroplasty Register have improved over the years. Possible reasons for this include better surgical technique with triceps-sparing approaches, better cementing technique and equipment, and a postoperative regime that allows the triceps to heal. Also, it is possible that the elbows that underwent earlier operations may have undergone more severe destruction from RA, and were thus less optimal for implant arthroplasty.

According to our results, it appears that success of reconstruction of a non-weight bearing joint such as the elbow by means of TEA is not as affected by implant choice to the same extent as hip (Eskelinen et al. 2005) and knee (Robertsson et al. 2001) arthroplasty. The explanation for this may be that both the contemporary semi-constrained and unconstrained TEA designs perform in a more or less unconstrained manner when properly inserted (Hargreaves and Emery 1999).
